# Immune-Mediated Adverse Events Associated with Ipilimumab CTLA-4 Blockade Therapy: The Underlying Mechanisms and Clinical Management

**DOI:** 10.1155/2013/857519

**Published:** 2013-04-17

**Authors:** Ahmad Tarhini

**Affiliations:** Division of Hematology/Oncology, Department of Medicine, University of Pittsburgh Cancer Institute, UPMC Cancer Pavilion, 5150 Centre Avenue, Room 555, Pittsburgh, PA 15232, USA

## Abstract

Immunomodulation with the anti-CTLA-4 monoclonal antibody ipilimumab has been shown to extend overall survival (OS) in previously treated and treatment-naive patients with unresectable stage III or IV melanoma. Blockade of CTLA-4 signaling with ipilimumab prolongs T-cell activation and restores T-cell proliferation, thus amplifying T-cell-mediated immunity and the patient's capacity to mount an effective antitumor immune response. While this immunostimulation has unprecedented OS benefits in the melanoma setting, it can also result in immune-mediated effects on various organ systems, leading to immune-related adverse events (irAEs). Ipilimumab-associated irAEs are common and typically low grade and manageable, but can also be serious and life threatening. The skin and gastrointestinal tract are most frequently affected, while hepatic, endocrine, and neurologic events are less common. With proper management, most irAEs resolve within a relatively short time, with a predictable resolution pattern. Prompt and appropriate management of these irAEs is essential and treatment guidelines have been developed to assist oncologists and their teams. Implementation of these irAE management algorithms will help ensure that patients are able to benefit from ipilimumab therapy with adequate control of toxicities.

## 1. Introduction

Melanoma is considered an “immunogenic” tumor—a theory that is supported by several observations and reported in the literature. The host immune system actively responds to melanoma, where advanced malignancy has rarely been reported to undergo spontaneous regression [1–4]. This may also be supported by the presence of lymphoid infiltrates at the site of primary melanoma associated with pathologic evidence of tumor regression. T-cell infiltration in primary melanoma was shown to be independently prognostic of improved survival [5]. Moreover, the absence of tumor infiltrating lymphocytes (TILs) at the primary melanoma site was associated with a higher probability of sentinel lymph node (SLN) melanoma metastasis compared with brisk TILs [6]. In patients treated with interferon-*α*2b (INF*α*2b), T-cell infiltration within nodal metastasis was associated with benefit from INF*α*2b therapy [7–9]. Clinically, this is also supported by the fact that for three decades, the only two therapeutic agents approved by the US Food and Drug Administration for the treatment of melanoma were immunotherapeutic (adjuvant IFN*α* and high-dose interleukin-2). 

Until recent regulatory approvals, patients with advanced melanoma have had few therapeutic options [27], and conventional treatment was limited by low tumor response rates, poor patient survival outcomes, and toxicity [28–32]. As such, several immunotherapeutic approaches, such as agents targeting immune checkpoints, have been developed and are under evaluation as antimelanoma interventions [33]. One of these—YERVOY (ipilimumab)—is an anticytotoxic T-lymphocyte-associated antigen (CTLA)-4 antibody that augments T-cell activation and proliferation. In 2011, ipilimumab was approved by the FDA for all patients with unresectable or metastatic melanoma, and by the European Medicines Association (EMEA) for adult patients with previously treated advanced melanoma.

In phase III studies, adding ipilimumab to experimental or conventional therapy has been shown to extend overall survival (OS) in previously treated and treatment-naive patients with unresectable stage III or IV melanoma [16, 17]. The most common safety events associated with ipilimumab are immune-related adverse events (irAEs), which reflect the mechanism of action of ipilimumab. These events are dose dependent, schedule related, and cumulative [18, 34–37], and most frequently affect the skin and gastrointestinal (GI) tract [24]. This paper discusses irAEs associated with ipilimumab therapy and their underlying mechanisms, while also providing guidance for their management.

## 2. Tumor Immunology and Immune Checkpoints

The immune system plays an important role in identifying and eliminating tumors. Transformed tumor cells expressing tumor-associated antigens (TAAs) were not found on normal cells [38]. These TAAs are recognized as “not-self” by the immune system, and T cells can be activated in response to cellular presentation of TAAs. T-cell activation is a tightly regulated process that requires two signals [39–42]. TAAs presented in context with the major histocompatibility complex (MHC) I or II on specialized antigen-presenting cells (APCs) bind with T-cell receptors (TCRs). Translation of TCR stimulation into T-cell activation requires a costimulatory signal in which B7 molecules on the APC surface bind with CD28 receptors on the T-cell surface. This induces T-cell proliferation, cytokine secretion, and changes in gene expression and metabolism. Activated T cells and antibodies against TAAs are found in blood for numerous types of cancer [43].

However, while this T-cell activity can protect the host from the development of cancer, it can also alter tumor progression by promoting the expansion of tumor cells with decreased sensitivity to immune attack, leading to evasion of the immune system or the development of host tolerance [39–42, 44–46]. Furthermore, tumors have developed several other defenses to escape immune recognition, including delivery of inhibitory signals, expansion of myeloid-derived suppressor cells that suppress T-cell responses, and induction of immunosuppressive regulatory T cells (Tregs) [47–49]. 

Tumors may also use immune-checkpoint pathways as a mechanism of immune resistance, particularly against T cells that are specific for TAAs. These pathways maintain self-tolerance and modulate immune responses in peripheral tissues to reduce damage to normal tissue [23]. Many of these checkpoints are controlled by ligand-receptor interactions. Two well-studied immune-checkpoint receptors are CTLA-4 (also known as CD152) and programmed cell death protein 1 (PD-1; also known as CD279). Although both are inhibitory receptors, they regulate immune responses at different levels and by different mechanisms [10, 22, 41, 50–52]. CLTA-4 is a negative regulator of T-cell-mediated antitumor immune responses. Upon TCR stimulation, T-cell expression of CTLA-4 is upregulated. This molecule competes with CD28 for binding to B7 on APCs, preventing the costimulatory signal and blunting T-cell activation and proliferation via a homeostatic feedback loop. In physiologic conditions, this prevents autoimmunity and allows establishment of tolerance to self-antigens [53]. PD-1 acts to limit the activity of T cells in peripheral tissues during inflammatory response to infection and to limit autoimmunity [51, 52]. As such, PD-1 represents a substantial mechanism of immune resistance within the tumor microenvironment [22].

**Figure 1:**
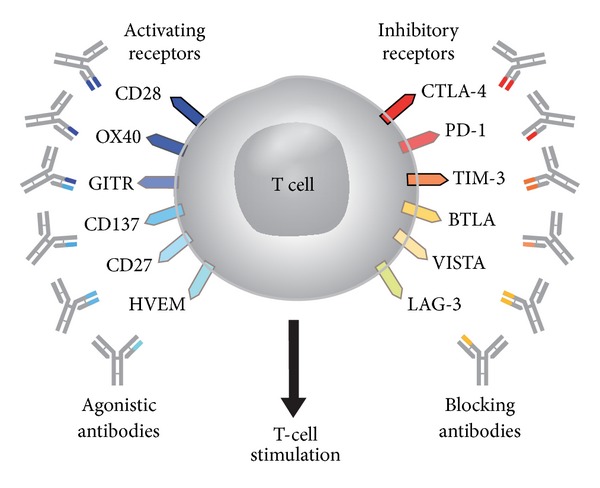
T-cell targets for immunoregulatory antibody therapy [10]; reproduced with permission from Mellman et al. 2011 [10].

Disruption of the native ligand-receptor interactions by exogenous antibodies, ligands, or receptors, allows immune checkpoints to be anticancer targets (Figure 1) [10]. The fully human monoclonal antibody ipilimumab competitively binds to CTLA-4 more efficiently than B7 while preserving CD28 signaling [37, 40, 54]. Blockade of CTLA-4 signaling prolongs T-cell activation and restores T-cell proliferation, thus amplifying T-cell-mediated immunity and the capacity of the patient to mount an effective antitumor immune response [55]. Recent data suggest that in patients with melanoma, ipilimumab can enhance both immune responses and humoral immunity mediated by different T-cell populations [56]. Blockade of other immune checkpoints may also be an anti-cancer strategy. A recent report in 207 patients indicates that antibody-mediated blockade of PD-1 induced durable tumor regression and prolonged stabilization of disease in patients with advanced cancers, including non-small-cell lung cancer, melanoma, and renal-cell cancer [57]. Another immune checkpoint protein worthy of note is CD137, which is expressed by activated T cells. Cross-linking of CD137—either by CD137-ligand binding or anti-CD137 antibody ligation—delivers a costimulatory signal to enhance T-cell activation and proliferation [58]. Preclinical studies have demonstrated that anti-CD137 antibodies can induce an antitumor response [59, 60] and clinical trials are under way [33, 61]. Understanding the immunostimulatory mechanism of action of ipilimumab provides important insight into the novel toxicity profile seen with this agent in the clinical setting. 

## 3. Efficacy of Ipilimumab Immunotherapy in Advanced Melanoma

Ipilimumab has been evaluated across a spectrum of patients with locally advanced unresectable or metastatic melanoma, including those who were heavily pretreated and those who were treatment naive (summarized in Table 1) [16–21]. 

Ipilimumab monotherapy at a dose of 3 mg/kg or 10 mg/kg every 3 weeks for four cycles (induction therapy) followed by maintenance therapy every 3 months has demonstrated anticancer activity in three phase II studies, with durable responses reported in around 80% of those patients who achieved clinical benefit (complete remission, partial remission, or stable disease) [18–20]. Another phase II study was undertaken to assess synergistic potential of ipilimumab combined with dacarbazine (DTIC) in advanced melanoma [21]. In this study, chemotherapy-naive patients were randomized to receive ipilimumab at 3 mg/kg every 4 weeks for four cycles either alone (*n* = 37) or with up to six 5-day courses of dacarbazine at 250 mg/m^2^/day (*n* = 35). Response rates and OS were improved in patients receiving combination therapy, suggesting that DTIC may provide additional benefit when added to ipilimumab; however, it should be noted that the patients in the ipilimumab monotherapy group had characteristics suggestive of poorer prognosis at baseline, which may have confounded study results.

More recently, ipilimumab has demonstrated efficacy in patients with advanced melanoma in two randomized, multicenter, phase III trials, both of which had OS as the primary endpoint (Table 1). In the first study, previously treated patients with unresectable stage III or IV melanoma were randomized to receive ipilimumab 3 mg/kg every 3 weeks for four cycles in combination with the experimental glycoprotein 100 (gp100) peptide vaccine (*n* = 403), ipilimumab 3 mg/kg every 3 weeks for four cycles alone (*n* = 137), or gp100 alone (*n* = 136) [16]. Eligible patients were also required to be HLA-A*0201-positive, based on the mechanism of action of the gp100-peptide vaccine that is HLA-A*0201 restricted. Compared with gp100 alone, OS was significantly extended with ipilimumab plus gp100 (hazard ratio (HR) for death 0.68; *P* < 0.001) and ipilimumab alone (HR for death 0.66; *P* = 0.003). Median OS in the ipilimumab groups was approximately 10 months, which was notable in this study population with poor prognosis. In this trial, patients with stable disease for a duration of 3 months after week 12 or a confirmed partial or complete response were offered reinduction therapy with their assigned treatment regimen if they had disease progression. Thirty-one patients who initially received ipilimumab either alone or with gp100 underwent reinduction therapy with ipilimumab. Of these, six (21%) achieved an objective response following ipilimumab reinduction, while 15 (48%) achieved stable disease. The demonstration of survival benefit achieved with ipilimumab at 3 mg/kg led to this dose regimen being approved for the treatment of metastatic melanoma. Of note, retrospective analysis of 453 treatment experienced patients from four phase II studies who were either HLA-A*0201-positive or HLA-A*0201-negative and received ipilimumab demonstrated similar activity for ipilimumab, regardless of HLA-A*0201 status [62].

In the second study, treatment-naive patients with unresectable stage III or IV melanoma received either ipilimumab in combination with DTIC (*n* = 250) or DTIC alone (*n* = 252) [17]. Ipilimumab was administered at the experimental dose of 10 mg/kg every 3 weeks for four cycles as induction therapy. Patients with stable disease or an objective response and no dose-limiting toxic effects received ipilimumab or placebo every 12 weeks thereafter as maintenance therapy. Addition of ipilimumab to DTIC significantly improved OS versus DTIC alone (HR for death 0.72; *P* < 0.001), with higher OS rates in the combination therapy group at 1, 2 and 3 years.

Ipilimumab not only has been shown to increase median OS relative to other treatments at a study population level, but also has induced unusually long-lasting survival in individual patients. A recent report in 36 heavily pretreated patients with metastatic melanoma found that 3 out of 30 patients achieving complete remission with ipilimumab 10 mg/kg had ongoing remission at 36+, 34+, and 41+ months [63]. In addition, median duration of response was 16 months in the 11 patients who achieved disease control. In another analysis of 177 patients, all but one of the 15 complete responders had ongoing responses at 54+ to 99+ months [64]. Responses have also been achieved with ipilimumab in patients with brain metastases from melanoma, particularly when metastases are small and asymptomatic [65, 66].

Because of the immunologic mechanism of action of ipilimumab, clinicians have observed predictable patterns of response that differ from those observed with conventional chemotherapy or radiotherapy, which may be a reflection of the time required to mount an effective antitumor immune response [67, 68]. These novel patterns include shrinkage in baseline lesions without new lesions, durable stable disease (followed by a slow, steady decline in total tumor burden in some patients), response after an increase in total tumor burden, and response in the presence of new lesions—which might be perceived mistakenly as disease progression. All response patterns have been associated with favorable survival [67] and indicate that confirmation of true disease progression is essential prior to discontinuation of ipilimumab therapy. Specific immune-related response criteria (irRC) have been developed that expand conventional World Health Organization (WHO) and Response Evaluation Criteria In Solid Tumors (RECIST) criteria to account for differences in response kinetics between cytotoxic and immunotherapeutic agents [67].

## 4. Adverse Event Profile of Ipilimumab Immunotherapy in Advanced Melanoma

As CTLA-4 plays a pivotal role in regulating tolerance to self-antigens, CTLA-4 blockade with ipilimumab can result in autoimmune damage of various organ systems, leading to irAEs [34, 69, 70]. This is borne out by data from experimental models [71, 72] and clinical observations [13, 15, 35]. A pooled analysis of 14 phase I-III studies evaluating various doses of ipilimumab demonstrated that 64.2% of patients experienced an irAE of any grade (Table 2) [24]. The majority of irAEs were mild-moderate (grade 1-2) with death due to irAEs occurring in <1% of patients. The skin and GI tract were most frequently affected, while hepatic, endocrine, and neurologic events were less common [24].

The incidence and severity of irAEs associated with ipilimumab administration appear to be dose related. In a phase II trial comparing three dose levels of ipilimumab (0.3 mg/kg, 3.0 mg/kg, and 10 mg/kg) in patients who were pretreated for advanced melanoma—followed by maintenance in patients achieving an objective response or stable disease—the incidence of irAEs was 26%, 56%, and 70%, with an occurrence of grade 3-4 irAEs in 0%, 7%, and 25% of patients, respectively [18]. The respective incidences of AEs that led to drug discontinuation were 13%, 10%, and 27%. 

Ipilimumab-induced AEs and irAEs occurred at similar frequencies among patients in the phase II and III trials, regardless of HLA-A*0201 status [62]. However, in the phase III trial comparing ipilimumab plus DTIC with DTIC alone, there were some differences in grade 3-4 AEs compared with other ipilimumab studies. The overall incidence of grade 3-4 AEs was higher with ipilimumab plus DTIC compared with DTIC alone (56% versus 28%), as the rate of irAEs (38% versus 4%) [17]. Moreover, hepatic toxicity was increased with the addition of DTIC to ipilimumab compared with DTIC alone (overall incidence of transaminase elevation 29–33% versus 6%), possibly due to inherent hepatotoxicity of DTIC [73, 74]. Although rates of rash were consistent with expectations, there were lower rates of GI complications with no GI perforations and lower rates of endocrinopathy [17].

In the registrational phase III trial of ipilimumab and gp100, there were 14 deaths overall: eight in the ipilimumab plus gp100 arm, four in the ipilimumab alone arm, and two in the gp100 alone arm [16, 75, 76]. Seven of these 14 were considered to be immune related and the deaths, which have been attributed to ipilimumab, involved the GI tract (bowel perforation, enterocolitis, and liver failure) and the nervous system (Guillain-Barré syndrome). These data highlight the importance of timely and appropriate identification and management of irAEs in patients treated with ipilimumab.

### 4.1. Timing and Resolution of irAEs Across Ipilimumab Phase II and III Studies

The onset of irAEs can be rapid and typically observed during the induction period of ipilimumab treatment. In the phase III registrational study comparing ipilimumab with gp100, 86% grade 2–5 irAEs occurred within the first 3 months of treatment [25]. However, in some cases, irAEs have occurred in patients many weeks or even months after receiving the last dose of therapy [25, 77]. 

**Figure 2:**
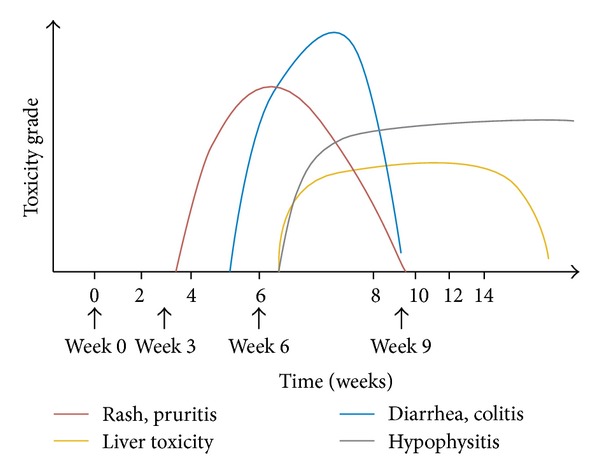
Kinetics of appearance of irAEs according to organ system involved [11]; adapted with permission from Weber et al. 2012 [11].

The time to onset and resolution of irAEs varies according to the organ system involved. Dermatologic irAEs are often evident after 2 to 3 weeks, GI and hepatic AEs after 6 to 7 weeks, and endocrinologic AEs only after an average of 9 weeks (Figure 2) [11, 77]. In the phase III registrational study, 88% of grade 2–4 irAEs resolved within 3 months (Table 3) [25]. Skin, GI, and liver events typically resolve within a few weeks, whereas endocrine events can take approximately 20 weeks to resolve, and are irreversible in some cases. Overall, time to onset (5-6 weeks) and time to resolution (4–8 weeks) of events are similar for the 3-mg/kg approved dose of ipilimumab and the investigational higher dose of 10 mg/kg [25]. Importantly, data from a pooled analysis of 325 patients with metastatic melanoma treated four times with ipilimumab 10 mg/kg once every 3 weeks showed that with the exception of hypophysitis (for which the irAE peaked and remained at grade 3 at week 14), other irAEs, such as rash, pruritus, and diarrhea, and colitis and liver toxicity all resolved to grade 0 or 1 by weeks 10 and 14, respectively [11].

### 4.2. Specific irAEs with Ipilimumab in Phase II and III Studies

#### 4.2.1. Dermatologic irAEs

**Figure 3:**
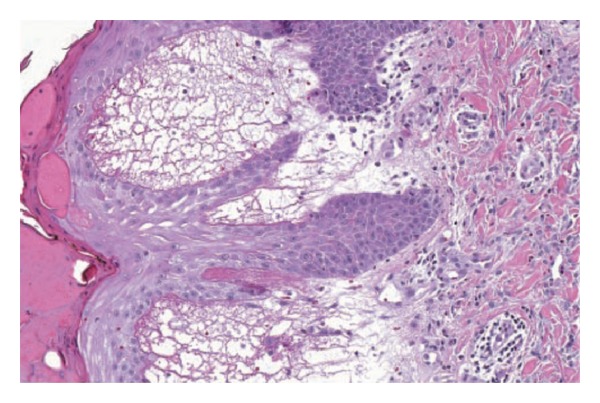
Skin biopsy showing severe dermatitis with epidermal spongiosis, papillary dermal edema, and a prominent inflammatory infiltrate in both the superficial and deep dermis [12]; reproduced with permission from Phan et al. 2003 [12].

Ipilimumab-induced dermatologic irAEs typically presented as a rash that was usually maculopapular, often accompanied by significant, generalized pruritus, and appeared to differ from that observed with tyrosine kinase inhibitors [78–80]. Biopsies showed severe dermatitis with papillary dermal edema, sometimes accompanied by perivascular lymphocytic infiltrate (Figure 3) [15]. Immunohistochemical staining of biopsied samples showed the presence of CD4+ and melan-A-specific CD8+ T cells in close proximity to apoptotic melanocytes, suggesting that anti-CTLA-4 antibodies stimulated an immune response directed against melanocytes [11]. As such, vitiligo and depigmentation—and also alopecia—have been reported with ipilimumab therapy [69, 80, 81].

**Figure 4:**
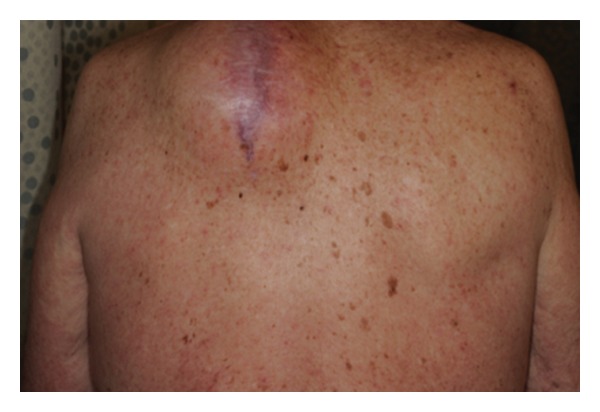
Immune-related maculopapular rash in a patient receiving ipilimumab.

In phase II and III ipilimumab trials, dermatologic irAEs were the most frequently reported AEs (Figure 4), occurring in around 65% of patients; however, these were typically mild to moderate in severity, with <3% being grade 3 or higher [24]. In the registrational phase III study, 40% and 43.5% of patients receiving ipilimumab plus gp100 and ipilimumab alone, respectively, had a dermatologic irAE; of these, 2.1% and 1.5%, respectively, were grade 3 or higher [16]. Severe, life-threatening, or fatal immune-mediated dermatitis (Stevens-Johnson syndrome, toxic epidermal necrolysis, or rash complicated by full thickness dermal ulceration, or necrotic, bullous, or hemorrhagic manifestations; grade 3–5) occurred in 13 of 511 (2.5%) patients treated with ipilimumab. One patient (0.2%) died as a result of toxic epidermal necrolysis, and one additional patient required hospitalization for severe dermatitis [75].

#### 4.2.2. Gastrointestinal irAEs

**Figure 5:**
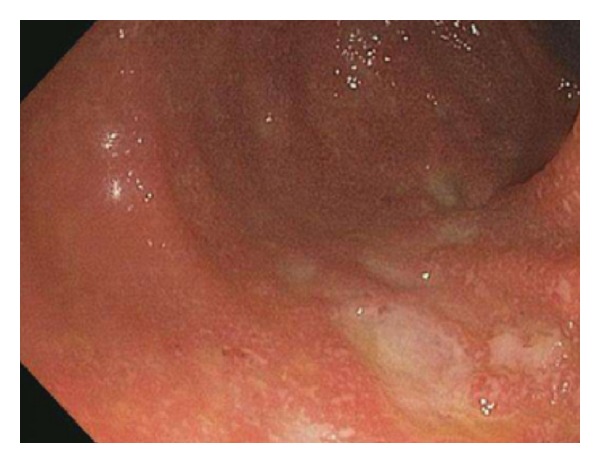
Ulcerated colonic mucosa, as viewed by colonoscopy, in a patient experiencing ipilimumab-related colitis.

**Figure 6:**
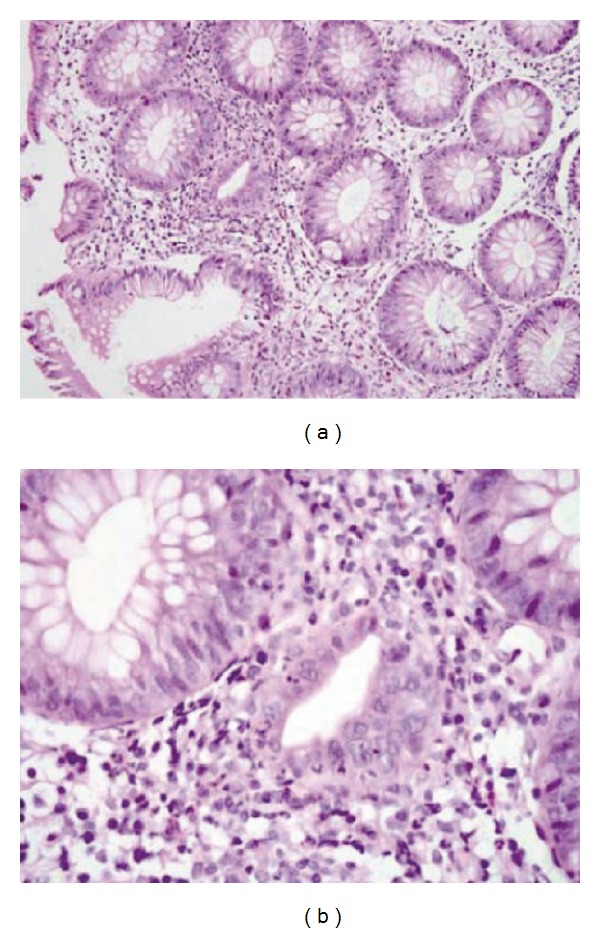
Histopathologic analyses showing focal active colitis (a) with crypt destruction, loss of goblet cells, and neutrophilic infiltrates in the crypt epithelium (b) [13]; reproduced with permission from Maker et al. 2005 [13].

CTLA-4 blockade with ipilimumab can cause dysregulation of GI mucosal immunity that results in irAEs such as diarrhea and colitis, or events that involve the esophagus, duodenum, ileum, and stomach [42, 82]. This dysregulation of GI mucosal immunity is likely to be a distinct clinicopathologic entity that differs from that seen with inflammatory bowel diseases [83]. Patients may present with diarrhea, abdominal pain, blood in stools, increased stool frequency, nausea, vomiting, or constipation, with or without fever. Extensive ulcerations observed by colonoscopy (Figure 5) indicate severe cases, but a mild presence of colitis on macroscopic evaluation could be misleading, as biopsies with severe inflammation often occur in the presence of mild macroscopic changes. Colitis may be associated with other GI complications, such as aphthous ulcers, esophagitis, gastritis, and jejunitis [34, 37], and can demonstrate a diverse range of inflammatory histopathologies [34, 84–86]. In one report, histopathologic analysis showed focal active colitis with crypt destruction, loss of goblet cells, and neutrophilic infiltrates in the crypt epithelium (Figure 6) [13]. Similarly, neutrophilic, lymphocytic, and mixed neutrophilic-lymphocytic infiltrates have been previously observed in 46%, 15%, and 38% of patients, respectively [11, 87]. Together, these histological reports provide potential mechanisms of action for the irAEs associated with ipilimumab administration. It should be noted that diarrhea and/or colitis can become life threatening [21, 34, 84, 88, 89] with reports of fatal bowel perforation and sepsis [21, 88, 90]. 

A retrospective review of safety data from 14 completed phase II and III trials of 1498 patients treated with ipilimumab showed that GI irAEs occurred in around 33% of patients, with nearly 10% affected by events grade 3 or higher [24]. Of these 1498 patients, three died due to GI irAEs [24]. In the registrational phase III study, 32.1% and 29% of patients receiving ipilimumab plus gp100 and ipilimumab alone, respectively, had a GI irAE; of these, 5.8% and 7.6%, respectively, were grade 3 or higher [16]. Across a review of 511 patients treated with ipilimumab, 5 patients (1%) developed intestinal perforation, 26 patients (5%) were hospitalized for severe enterocolitis [75], and 4 patients (0.8%) died as a result of complications related to enterocolitis [16, 75].

#### 4.2.3. Endocrine irAEs

Endocrine irAEs are diverse, associated with a range of nonspecific symptoms and, although less common, can be life threatening [91]. Endocrine irAEs consist of hypothyroidism and hyperthyroidism secondary to thyroiditis, hypopituitarism, hypophysitis, adrenal insufficiency, and hypogonadism. The most common clinical presentation includes headache and fatigue. Symptoms may also include visual field defects, behavioral changes, decreased libido, electrolyte disturbances, and hypotension [75]. Prolonged exposure to corticosteroid therapy, possibly to manage other irAEs, may also lead to adrenal insufficiency and hypogonadism, and should be taken into consideration during the assessment of endocrinopathies in these patients. 

**Figure 7:**
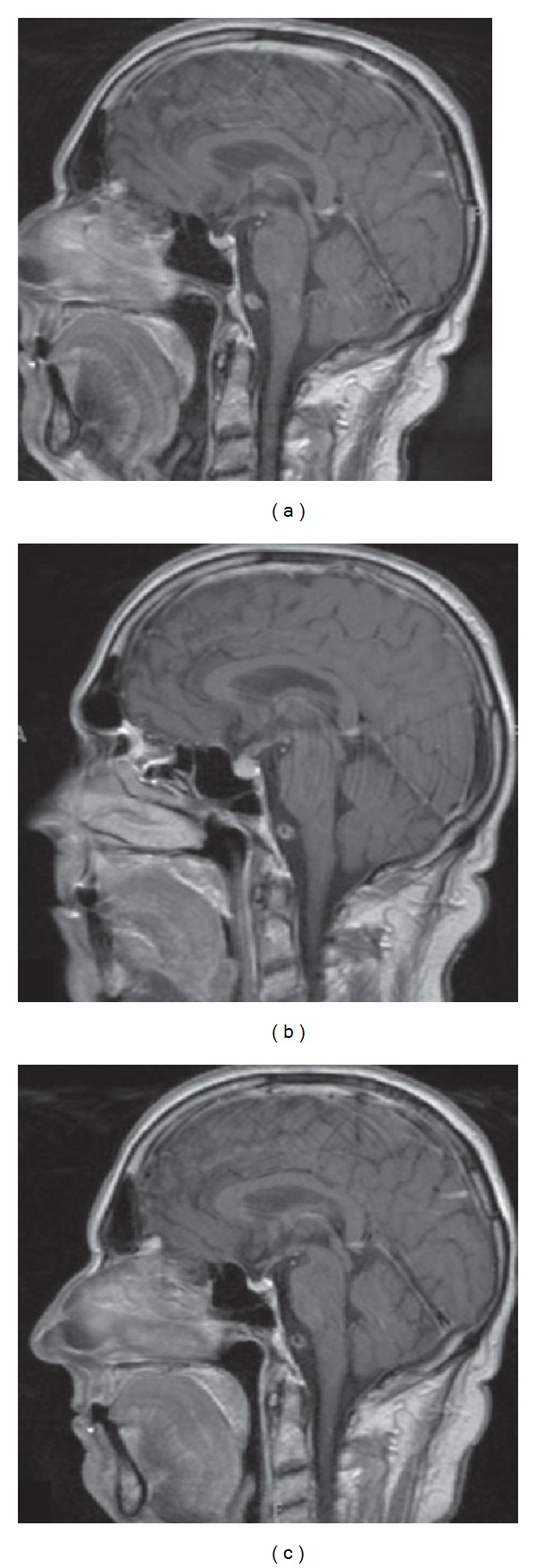
Magnetic resonance images of the brain demonstrating ipilimumab-associated hypophysitis. (a) Prior to therapy, with no metastatic disease indicated. (b) Diffuse enlargement of the pituitary gland following reports of cognitive impairment during therapy. (c) Resolution of hypophysitis after discontinuation of ipilimumab and initiation of hormone-replacement therapy [14]; reproduced with permission from Carpenter et al. 2009 [14].

Hypophysitis is the most commonly reported endocrine irAE associated with ipilimumab [34], and lymphocytic hypophysitis has been reported in 0–17% of patients in trials of ipilimumab [35, 92]. It is presumed to be secondary to a lymphocytic infiltration of the pituitary leading to enlargement of the gland, followed by damage to the pituitary cells with hypofunction of ACTH, TSH, and other secreting cells leading to secondary adrenal insufficiency and hypothyroidism. The imaging characteristics of hypophysitis are also non-specific and, on the basis of imaging alone, often cannot be differentiated from other causes, including metastasis [14]. Clinically, affected patients present with symptoms and signs typical of a pituitary mass effect, such as headache, and possibly visual disturbances, lethargy, nausea, fatigue, and loss of libido. Laboratory tests may show low ACTH, cortisol, TSH, free T4, and electrolyte abnormalities. MRI findings usually show homogeneous enhancement and uniform enlargement of the pituitary gland, although the enlargement is relatively modest (Figure 7). Loss of posterior pituitary signal intensity on precontrast MR images has commonly been reported, as well as a variable enlargement of the infundibulum. 

The incidence of endocrine irAEs in ipilimumab phase II and III trials of 1498 patients was <5%, with <3% being grade 3 or higher [24]. In the registrational phase III study, 3.9% and 7.6% of patients receiving ipilimumab plus gp100 and ipilimumab alone, respectively, had an endocrine irAE; of these, 1.1% and 3.8%, respectively, were grade 3 or higher [16]. Endocrine irAEs consisted of hypothyroidism, hypopituitarism, hypophysitis, adrenal insufficiency, increased levels of serum TSH, and decreased levels of serum CRH.

#### 4.2.4. Hepatic irAEs

Inflammatory hepatitis similar to autoimmune disease, including severe and fatal cases, has been reported in patients receiving ipilimumab. It is important to rule out other etiologies (e.g., infection, metabolic, alcohol abuse). Patients may develop elevated alanine aminotransferase, aspartate aminotransferase, and/or hyperbilirubinemia in the absence of clinical symptoms. Importantly, biopsies from patients experiencing immune-related hepatotoxicity showed diffuse T-cell infiltrate consistent with immune-related hepatitis [11]. The incidence of hepatic irAEs in ipilimumab phase II and III trials of 1498 patients was <2%, with around 1% (*n* = 16) being grade 3-4 in severity. Of 1498 patients, two deaths were attributed to hepatic irAEs [24]. One of these fatalities was a treatment-related death by liver failure and occurred in a patient receiving ipilimumab 10 mg/kg who did not promptly receive systemic corticosteroids [20]. In the registrational phase III study, 2.1% and 3.8% of patients receiving, respectively, ipilimumab plus gp100 and ipilimumab alone had a hepatic irAE; of these, 1.1% and 0%, respectively, were grade 3 or higher [16]. Increase in alanine aminotransferase and aspartate aminotransferase was also reported [16]. In the second phase III trial, the combination of ipilimumab and DTIC led to increased hepatotoxicity compared with DTIC alone [17].

#### 4.2.5. Ocular irAEs

**Figure 8:**
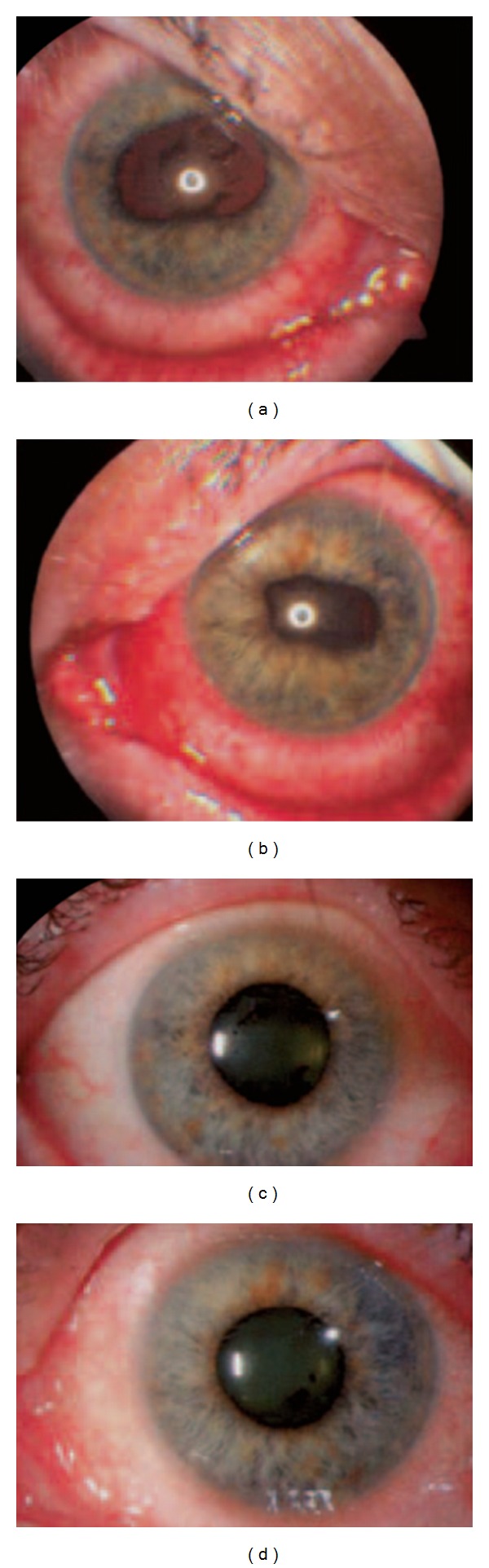
Ophthalmologic images of ipilimumab-associated uveitis in both eyes. (a, b) At the time of presentation, with irregular pupils caused by iris adhesions to the lens; (c, d) the same patient after 4 days of topical corticosteroid therapy [15]; reproduced with permission from Attia et al. 2005 [15].

Ocular events that have been reported with ipilimumab include conjunctivitis, scleritis [93], uveitis [75, 93], and Graves' ophthalmopathy [94, 95]. Ophthalmologic photographs from a patient with ipilimumab-induced uveitis are provided in Figure 8. Ocular inflammation is usually observed in association with colitis and therefore, it is recommended that all patients experiencing colitis undergo an ophthalmological evaluation. An ophthalmologist should evaluate visual complaints with examination of the conjunctiva, anterior and posterior chambers, and retina; visual field testing and an electroretinogram should also be performed. The incidence of ocular irAEs in ipilimumab phase II and III trials was 1.3%, with 0.4% being grade 3 or higher [24].

#### 4.2.6. Neurologic irAEs

Patients usually present with muscle weakness or sensory neuropathies lasting several days, or motor neuropathies confirmed by physical examination. Neurologic irAEs associated with ipilimumab include inflammatory myopathy [96], aseptic meningitis with cerebrospinal fluid lymphocytosis [97], severe meningo-radiculo-neuritis [98], temporal arteritis [20], and Guillain-Barré syndrome [99]. Inflammatory enteric neuropathy with severe constipation has also been reported after ipilimumab treatment [100], as posterior reversible encephalopathy syndrome [101]. Myasthenia gravis-like symptoms have also been reported in <1% of patients who received higher doses of ipilimumab in clinical trials [75]. 

The incidence of neurologic irAEs in ipilimumab phase II and III trials was 0.1%, with no grade 3-4 events [24]. However among the 1498 patients included in the analysis, one death (<0.1%) was recorded in a patient in the registrational phase III trial due to Guillain-Barré syndrome [16].

#### 4.2.7. Other irAEs

Other irAEs reported with ipilimumab therapy include autoimmune pancreatitis [15, 102], red cell aplasia [103], pancytopenia [104], and autoimmune neutropenia [105]. Sarcoidosis [106–108], systemic vasculitis, including kidney disease [20, 109], and acquired hemophilia A due to the presence of a factor VIII inhibitor [110, 111] have also been reported.

Additional AEs suspected to be immune related, which were reported in <2% of patients receiving ipilimumab alone in the registrational phase III trial, included eosinophilia, lipase elevation, and glomerulonephritis. Iritis, hemolytic anemia, amylase elevations, multiorgan failure, and pneumonitis were reported in patients receiving ipilimumab in combination with gp100 [75].

### 4.3. Impact of irAEs on Clinical Benefit of Ipilimumab

Early ipilimumab trials suggested an association between development of irAEs and clinical benefit [11, 12, 100, 112]. A similar observation was made in patients treated with the combination of tremelimumab and IFN-*α* during a clinical trial [113]. Although radiologic manifestations of irAEs—such as those clinically evident (colitis, hypophysitis, and arthritis) and those clinically silent (benign lymphadenopathy and inflammatory changes in soft tissue), all assessed by the standard methods of tumor imaging—are associated with significant clinical benefit of anti-CTLA-4 therapy [114], the potential link between irAEs and clinical benefit awaits further confirmation and clarification.

An immune-active tumor microenvironment appears to favor clinical response to ipilimumab [115]. In a recent report, patients with high pretreatment expression levels of immune-related genes were more likely to respond favorably to ipilimumab. Furthermore, ipilimumab appeared to induce two major changes in tumors from patients who exhibited clinical activity: genes involved in immune response showed increased expression, whereas expression of genes for melanoma-specific antigens and genes involved in cell proliferation decreased [115]. These changes were associated with the total lymphocyte infiltrate in tumors, and there was a suggestion of association with prolonged OS. Many IFN-*γ*-inducible genes and Th1-associated markers showed increased expression after ipilimumab treatment, suggesting an accumulation of this particular type of T cell at tumor sites, which might play an important role in mediating the antitumor activity of ipilimumab [115]. These data may play an important role in the efforts to develop therapeutic predictive biomarkers for ipilimumab that would allow the specification of therapy for those patients who are most likely to benefit, while saving others from unwanted toxicities in the absence of predicted benefit. 

## 5. Management of Ipilimumab-Associated irAEs

Given the nature of irAEs, which differ from the AEs typically seen with conventional cytotoxic chemotherapy, algorithms for the management of GI irAEs, diarrhea, endocrine, hepatic irAEs, and neurologic were developed and used in clinical trials with ipilimumab [77, 116]. Since then, a more comprehensive and standardized set of guidelines have been developed to manage irAEs that highlight vigilance and the use of corticosteroids or other immunosuppressants when appropriate (Table 4). Healthcare professionals can find the latest version of these guidelines here: https://www.hcp.yervoy.com/pages/rems.aspx/.

Of note, the use of corticosteroids to manage irAEs associated with ipilimumab does not appear to negatively impact the efficacy of ipilimumab [117–119], and duration of tumor response does not appear to be affected by the use of corticosteroids for abrogation of treatment-related toxicities [120]. A phase 2 study of ipilimumab monotherapy in advanced melanoma with brain metastases enrolled 72 patients, 51 of whom had asymptomatic brain metastases (i.e., no corticosteroids at study entry; cohort A) and 21 of whom had symptomatic brain metastases that were being managed through a stable dose of corticosteroids (cohort B) [66]. The study reported disease control in 9 patients (18%) and 1 patient (5%) in cohorts A and B, respectively, and disease control in the brain for 12 patients (24%) and 2 patients (10%), respectively. There were no unexpected toxicities in either cohort. Demonstration of some efficacy despite the small sample size in cohort B, which represents a population with a relatively very poor prognosis, does suggest that concurrent corticosteroids do not completely abrogate the potential for ipilimumab-mediated responses. It is not clear, however, the extent to which corticosteroid therapy influenced the observed differences in efficacy outcomes for the two cohorts, and further research is needed to better understand the impact of concurrent steroids on ipilimumab therapy [66]. Delaying the initiation of corticosteroids when indicated may lead to more serious AEs and complications that could be fatal. In general, the management of irAEs is dependent on severity. For example, grade 1-2 irAEs are treated symptomatically, with increased frequency of monitoring. Grade 1-2 irAEs that remain persistent should be managed as one would for grade 3-4 irAEs. And, grade 3-4 irAEs should be treated with corticosteroids and tapered over 4 or more weeks.

### 5.1. Dermatologic irAES

Mild or moderate itching with or without rash (grade 1 and 2) is generally managed with symptomatic therapy. Nonirritant moisturizers and body wash, thick moisturizing creams or ointments, low-dose topical corticosteroids (betamethasone 0.1% or hydrocortisone 1%) or urea-based topical therapies with antihistamines, such as diphenhydramine HCl or hydroxyzine HCl, should be employed [11, 121–123]. Sun avoidance and the use of broad-spectrum sunscreen are recommended. Cool compresses may also assist with symptomatic relief of pruritus. Scalp lesions may benefit from low-dose, corticosteroid-containing shampoo, or topical cold tar.

Persistent grade 1-2 Common Terminology Criteria for Adverse Events (CTCAE) symptoms should be treated with corticosteroids [26, 124]. Higher dose topical corticosteroids may be required for a more severe rash (hydrocortisone 2%), and oral prednisone 1 mg/kg/day should be initiated if there is no improvement or if the rash is associated with other dermal complications [121]. Grade 3-4 rash and intense pruritus should be evaluated by a dermatologist and require administration of systemic corticosteroids (e.g., prednisone 1 mg/kg/day) and drug interruption/discontinuation. It is recommended that patients with grade 3 dermatologic irAEs discontinue ipilimumab until symptoms resolve to grade 1 or less and systemic corticosteroid therapy has been safely tapered. Patients with grade 4 dermatologic irAEs should permanently discontinue ipilimumab. It should be noted that patients with grade 4 irAEs often require systemic IV corticosteroid therapy (e.g., methylprednisolone 1-2 mg/kg/day), tapering over not less than 30 days.

### 5.2. Gastrointestinal irAEs

Early initiation of diarrhea treatment guidelines has been shown to reduce bowel perforation and colectomy rates, drug-related diarrhea, and serious GI irAEs by up to 50% in patients treated with ipilimumab [125]. A complete work-up to rule out other causes of diarrhea, such as infections, should be initiated at the onset of diarrhea or colitis in all patients treated with ipilimumab. GI consultation and colonoscopy should be strongly considered in patients with persistent or severe symptoms. The objective of GI irAE resolution is to reverse the inflammation caused by the immune response and is based on the use of immunosuppressive agents. It is important to maintain frequent contact with the patient to monitor symptoms and any changes in symptoms to prevent further escalation in severity. Any reports of change in pain should be evaluated for the possibility of perforation, peritonitis, or pancreatitis. Caution should be exercised when using analgesics (e.g., morphine) to control abdominal pain, as they may mask symptoms of such severe complications.

Options for management of mild (grade 1) GI irAEs include symptomatic treatment with loperamide or diphenoxylate, oral hydration, and electrolyte substitution [11, 26, 124]. Moderate (grade 2) symptoms may be initially treated conservatively, but should be immediately switched to corticosteroids if symptoms persist or worsen (e.g., prednisone 1 mg/kg daily). Symptoms can also be treated initially with oral diphenoxylate hydrochloride and atropine sulfate four times per day and budesonide 9 mg once per day [11], or divided three times per day. In a randomized phase II study of ipilimumab, prophylactic budesonide did not decrease the rate of GI irAEs and is not recommended as monotherapy for grade ≥2 diarrhea associated with ipilimumab therapy [19]. 

In the case of grade 3 or 4 diarrhea, ipilimumab treatment should be discontinued [11, 26] and the patient should be treated with high-dose intravenous methylpredisolone (2 mg/kg for 1-2 weeks) followed by a taper lasting for a minimum of 30 days [121, 122]. A rapid reduction in corticosteroid dose should be avoided, as this may increase the risk of developing recurrent symptoms and the need for escalation of care [121]. Alternatively, a steroid regimen with intravenous dexamethasone 4 mg every 6 hours, initially over 7 days, is another treatment option [121]. If there is no improvement within 5–7 days, or relapse occurs, single-dose infliximab 5 mg/kg should be considered (unless contraindicated [e.g., presence of sepsis, other serious infections, perforation are present]) [121, 122]. An additional dose after a 2-week interval (possibly in combination with mesalamine, loperamide and hydrocortisone enemas) may be required [85, 121, 122]. The use of low doses of oral corticosteroids in patients with grade 3 diarrhea may be suboptimal and lead to serious complications.

GI infection or inflammatory bowel disease should be ruled out in all patients with persistent or severe diarrhea by examination for stool leukocytes, stool cultures, and a *Clostridium difficile* titer. Sigmoidoscopy or colonoscopy to confirm or rule out colitis with a GI consultation is indicated for persistent grade 2 or severe diarrhea or rectal bleeding [11].

### 5.3. Management of Endocrine irAES

If symptoms are suggestive of an endocrinopathy and the patient is not in adrenal crisis, endocrine laboratory results should be evaluated before corticosteroid therapy is initiated. Endocrine work-up should at least include TSH and free T4 levels to determine if thyroid abnormalities are present. TSH, prolactin, and a morning cortisol level will help to differentiate primary adrenal insufficiency from primary pituitary insufficiency. Grade 1 or 2 endocrine toxicity without adrenal crisis may resolve spontaneously if a patient has no or minimal symptoms, but should be monitored closely, possibly with guidance from an endocrinologist; otherwise, short-term, high-dose corticosteroids (e.g., dexamethasone 4 mg every six hours or equivalent) with relevant hormone replacement (e.g., levothyroxine, hydrocortisone, sex hormones) will be needed [26, 123, 124]. Patients with symptoms suggestive of hypophysitis, such as headaches, visual disturbances, polyuria, extreme thirst, and hyperprolactinemia, require prompt corticosteroid replacement while awaiting confirmation of diagnosis by MRI [122]. A short course of high-dose dexamethasone followed by physiologic hormone replacement has produced a partial recovery of pituitary function in some patients [126]; however, pituitary dysfunction may be permanent [122] and patients will need ongoing low-dose corticosteroid replacement therapy [26, 124]. Ipilimumab may be reinstituted after resolution of grade 1-2 endocrinopathies; however, because the risk of further complications is unknown, it is not recommended in more severe cases. Patients with hypophysitis with the need for hormone replacement have been retreated with ipilimumab without worsening side effects [121]. Patients with endocrine toxicity in adrenal crisis (characterized by a constellation of symptoms suggestive of severe dehydration, hypotension, or shock) should be treated as a medical emergency and given intravenous injections of glucocorticoids and large volumes of intravenous saline solution with dextrose. 

### 5.4. Management of Hepatic irAES

For grade 1-2 liver toxicity with ongoing symptoms, corticosteroids should be given and tapered over at least one month. Ipilimumab may be held until symptoms resolve. For grade 2 liver toxicity, ipilimumab should be withheld and oral corticosteroid therapy (prednisone 1-2 mg/kg/day) started for at least 30 days if there is no improvement after 48–72 hours [121]. Corticosteroid therapy and ipilimumab discontinuation are recommended for grade 3 AEs [121]. Grade 3-4 liver toxicity is treated with high-dose intravenous corticosteroid treatment (e.g., methylprednisolone 2 mg/kg once or twice daily) [26, 124]. If there is no improvement in transaminases after 48 hours, the addition of mycophenolate mofetil 1 g twice daily is recommended. If there is no improvement after a further 5–7 days, tacrolimus 0.10–0.15 mg/kg/day is recommended. One case report has also described successful treatment of ipilimumab-induced fulminant hepatitis with antithymocyte globulin (1.5 mg/kg given at 4 intervals over 2 weeks) [127]. Treatment with infliximab 5 mg/kg as a single dose has also been described as an option in refractory and severe cases [121]; however, currently this approach is not recommended because of the potential for severe hepatic reactions, including autoimmune hepatitis, in patients receiving infliximab [128]. 

### 5.5. Management of Ocular irAES

Ocular irAEs (such as uveitis) usually resolve spontaneously within a week; however, treatment with corticosteroid eye drops may be required [121, 122]. Systemic corticosteroids may be required in more severe cases [121].

### 5.6. Management of Neurologic irAES

Patients with grade 3-4 neurologic symptoms may require hospitalization, intravenous corticosteroids, intravenous immunoglobulin, or other immunosuppressants. Ipilimumab should be ceased in affected patients [121].

## 6. Concluding Remarks

Ipilimumab has shown significant improvements in OS in patients with advanced melanoma within clinical trials, and these benefits may extend for years in some patients. This potential for long-term survival comes at the cost of a toxicity profile that is atypical compared with other melanoma therapies, reflecting the immune-mediated mechanism of action of ipilimumab. Although common, ipilimumab-associated irAEs are typically low grade and manageable [129]. Moreover, with proper management, most AEs resolve within a relatively short timeframe, with a predictable resolution pattern [25]. Prompt and appropriate management of these irAEs is essential, and treatment guidelines have been developed to assist oncologists and their teams. Implementation of these irAE management algorithms will help ensure that patients are able to benefit from ipilimumab therapy with adequate control of toxicities. The potential of ipilimumab in the adjuvant and neoadjuvant settings is being explored; in this new era of targeted immunotherapy, the future for melanoma treatment is encouraging.

## Figures and Tables

**Table 1:** Ipilimumab efficacy in phase II and III trials [16–21].

Study	Phase	Population (*n*)	Treatment	ORR (%)	PFS	Median OS, months (95% CI)
CA184-022 [18]	II	Pretreated (*n* = 217)	Ipi 10 mg/kg Ipi 3 mg/kg Ipi 0.3 mg/kg	11.1% 4.2% 0.0%	24-week: 18.9% 24-week: 12.9% 24-week: 2.7%	11.4 (6.9–16.1) 8.7 (6.9–12.1) 8.6 (7.7–12.7)

CA184-008 [22]	II	Heavily pretreated, progressed on prior therapy (*n* = 155)	Ipi 10 mg/kg	5.8%	NR	10.2 (7.6–16.3)

CA184-007 [23]	II	Treatment naïve and previously treated (*n* = 115)	Ipi 10 mg/kg Ipi 10 mg/kg + budesonide^†^	15.8% 12.1%	NR	19.3 (12.0–34.5) 17.7 (6.8–45.0)

MDX010-08 [10]	II	Treatment naïve (*n* = 72)	Ipi 3 mg/kg* Ipi 3 mg/kg* + DTIC	5.4% 14.3%	NR	14.3 (10.2–18.8) 11.4 (6.1–15.6)

MDX010-20 [16]	III	Pretreated, progressed on prior therapy (*n* = 676)	Ipi 3 mg/kg + gp100 Ipi 3 mg/kg Gp100	5.7% 10.9% 1.5%	Median: 2.76 months Median: 2.86 months Median: 2.76 months	10.0 10.1 6.4

CA184-024 [17]	III	Treatment naïve (*n* = 502)	Ipi 10 mg/kg + DTIC DTIC	15.2% 10.3%	Median PFS similar in both arms, but overall 24% reduction in risk of progression for ipi + DTIC versus DTIC (HR 0.76; *P* = 0.006)	11.2 (9.4–13.6) 9.1 (7.8–10.5)

*Once every 4 weeks × 4 cycles (induction).

^†^Prophylactic budesonide was added to determine if the rate of grade ≥2 diarrhea was reduced.

CI: confidence interval; DTIC: dacarbazine; HR: hazard ratio; NR: not reported; ORR: objective response rate; PFS: progression-free survival; OS: overall survival; WHO: World Health Organization.

**Table 2:** Frequency of specific AEs* and irAEs in a pooled analysis of 1498 patients in phase I–III studies of ipilimumab in unresectable stage III or stage IV melanoma [24].

AEs (*n* = 1498)	Any grade, *n* (%)	Grade 3-4, *n* (%)	Grade 5, *n* (%)
Specific AE			
Diarrhea	554 (37.0)	104 (6.9)	0 (0)
Colitis	120 (8.0)	74 (4.9)	1 (<0.1)
Enterocolitis	18 (1.2)	9 (0.6)	0 (0)
Large intestine perforation	4 (0.3)	3 (0.2)	1 (<0.1)
Intestinal perforation	3 (0.2)	2 (0.1)	1 (<0.1)
Rash	498 (33.2)	37 (2.5)	0 (0)
Pruritus	413 (27.6)	6 (0.4)	0 (0)
Abnormal hepatic function	74 (4.9)	17 (1.1)	1 (<0.1)
Hepatitis	10 (0.7)	10 (0.7)	0 (0)
Hepatic failure	7 (0.5)	1 (<0.1)	5 (0.3)
Peripheral sensory neuropathy	67 (4.5)	6 (0.4)	0 (0)
Neuropathy peripheral	13 (0.9)	0 (0)	0 (0)
Peripheral motor neuropathy	9 (0.6)	6 (0.4)	0 (0)
Hypopituitarism	40 (2.7)	31 (2.1)	0 (0)
Hypothyroidism	27 (1.8)	2 (0.1)	0 (0)
Adrenal insufficiency	11 (0.7)	5 (0.3)	0 (0)
irAEs grouped by organ class			
Any irAE	962 (64.2)	266 (17.8)	9 (0.6)
Dermatologic	672 (44.9)	39 (2.6)	0 (0)
Gastrointestinal	487 (32.5)	137 (9.1)	3 (0.2)
Endocrine	68 (4.5)	34 (2.3)	0 (0)
Hepatic	24 (1.6)	16 (1.1)	2 (0.1)
Ocular	20 (1.3)	6 (0.4)	0 (0)
Neurologic	2 (0.1)	0 (0)	1 (<0.1)
Cardiovascular (myocarditis)	2 (0.1)	2 (0.1)	0 (0)

*Regardless of causality. Subjects may have had more than one event. Unknown intensities are included in “Any Grade” column. Grade 5 = death. Results from the following trials were included in the analysis: MDX010-02, -15, -03, -04, -13, -05, -19, -08, -20; CA184-042, -004, -008, -022, -007: These trials ranged from phase I–III, investigated ipilimumab at various doses (0.1–20 mg/kg), as monotherapy or in combination with various agents. All patients included had unresectable stage III or IV melanoma, no prior history or clinical evidence of autoimmune disease or treatment with immunosuppressive drugs, and received at least one dose of ipilimumab. Patient characteristics such as age, prior treatment history and performance status varied among trials. Safety events included in this analysis were those reported between first dose and 70 days after last dose of study therapy.

**Table 3:** Time to onset and resolution of irAEs in phase III and pooled phase II trials [25].

	Phase III MDX010-20 Ipilimumab 3 mg/kg (*n* = 131)	Pooled phase II data Ipilimumab 3 mg/kg (*n* = 111)
irAE time to onset		
Grade 2–5, *n*	45	38
Median, weeks (95% CI)	6.14 (3.71–8.14)	6.93 (4.86–7.57)
% of patients experiencing irAE within		
0–1 month	42	32
>1–3 months	44	63
>3–5 months	7	5
>5 months	7	0
irAE time to resolution		
Grade 2–4, *n*	44	38
Resolved, *n*	33	23
Median, weeks (95% CI)	6.86 (4.14–8.43)	5.71 (2.14–NR)
% of patients whose irAE resolved within		
0–1 month	52	74
>1–3 months	36	17
>3–5 months	9	9
>5 months	3	0

CI: confidence interval; irAE: immune-related adverse event; NR: not reached.

**Table 4:** General guidelines for recommended management of irAEs [26]. (adapted from YERVOY REMS 2011).

Site	Signs and symptoms	Management
Skin	Evaluate patients for signs and symptoms of pruritus, rash or other skin toxicities *Rule out non-irAE causes *	**Grade 1 or 2**: Mild to moderate, localized; papules/pustules covering <10%–30% of body surface (i) Topical thick moisturizers and oatmeal baths, antihistamines, topical corticosteroids should be added if no response (ii) If no improvement after 1-2 weeks with topical corticosteroids, treat with oral corticosteroid therapy (e.g., prednisone 1 mg/kg qd or equivalent) **Grade 3 or 4**: Intense or widespread >30%; skin sloughing <10%–30% of body surface; epidermal or mucus membrane detachment. (i) For grade 3 rash, potent topical corticosteroid creams may be used first, but systemic corticosteroids may be required (ii) Administer high-dose systemic corticosteroid therapy (e.g., prednisone 1-2 mg/kg PO or methylprednisolone 1-2 mg/kg IV or equivalent depending on severity, administered qd or bid); once symptoms are controlled, initiate corticosteroid taper ≥1 month (iii) Withhold ipilimumab dosing in patients with grade 3 or 4 skin toxicities (iv) Permanently discontinue ipilimumab in patients with grade 4 rash (e.g., Stevens-Johnson syndrome, toxic epidermal necrolysis or rash complicated by full thickness dermal ulceration or necrotic, bullous or hemorrhagic manifestations)

GI	Assess patients for changes in bowel habits and for the following signs and symptoms: diarrhea, abdominal pain, blood or mucus in stool with or without fever, peritoneal signs consistent with bowel perforation and ileus *Rule out infectious or alternate etiologies* A biopsy through endoscopy may be performed as clinically indicated; provide appropriate symptomatic treatment, and withhold or continue ipilimumab dosing as clinically appropriate	**Grade 1 or 2**: <4–6 stools/day over baseline, IV fluids <24 hours, abdominal pain, mucus or blood in stool (i) Symptomatic management (dietary modifications and loperamide or diphenoxylate) and increase frequency of monitoring (ii) Grade 2 may be initially treated with oral diphenoxylate hydrochloride and atropine sulfate four times per day and budesonide 9 mg once per day or divided three times per day (iii) If symptoms persist (5–7 days) or relapse, treat with corticosteroid therapy (e.g., prednisone 1 mg/kg qd or equivalent) **Grade 3 or 4**: 7 stools/day over baseline or more, IV fluids 24 hours, hospitalization, abdominal pain, fever, life-threatening consequences (i) Administer 1–2 mg/kg methylprednisolone or equivalent and then move forward with ensuring differential diagnosis (ii) Once diarrhea and other symptoms are controlled, corticosteroid dose should be gradually tapered over 1 month; if there is no improvement within 5 days, or relapse occurs after corticosteroids, administer a single dose of infliximab 5 mg/kg unless contraindicated; infliximab should not be used if perforation or sepsis is present; for patients with concomitant hepatitis, mycophenolate mofetil can be used instead (iii) Withhold ipilimumab dosing for moderate reactions until improvement to mild severity or complete resolution; for severe reactions (grade 3 or 4), permanently discontinue ipilimumab

Endocrine	Non-specific symptoms include fatigue, headache, changes in mental status, abdominal pain, unusual bowel habits and hypotension. Undertake appropriate work-up (TSH, free T4, ACTH and morning cortisol); consider co-syntropin stimulation test, LH, FSH, testosterone, and prolactin; radiographic imaging [e.g., MRI] with pituitary cuts should be performed to assess size of pituitary gland in patients with symptoms suggestive of hypophysitis)	**Endocrinopathy not suggestive of adrenal crisis** (i) For patients with abnormal endocrine work up, grade 1 or 2 endocrine toxicity without adrenal crisis may resolve spontaneously if a patient has no or minimal symptoms but should be monitored closely, possibly with guidance from an endocrinologist; otherwise, short-term, high-dose corticosteroids (e.g., dexamethasone 4 mg every six hours or equivalent) with relevant hormone replacement (e.g., levothyroxine, hydrocortisone, or sex hormones) (ii) Patients with symptoms suggestive of hypophysitis require prompt corticosteroid therapy **Adrenal crisis: severe dehydration, hypotension, or shock** (i) Immediately initiate intravenous corticosteroids with mineral corticoid activity (e.g., methylprednisolone) (ii) When condition stabilizes, initiate endocrine work up and start appropriate hormone replacement therapy **Moderate reactions or symptomatic endocrinopathy**(i) Withhold ipilimumab until complete resolution or stable on hormone replacement therapy (ii) Patients unable to have their corticosteroid dose reduced to 7.5 mg prednisone or equivalent per day should permanently discontinue ipilimumab (iii) Consider long-term hormone replacement therapy as necessary

Hepatic	Run LFTs before each infusion or more frequently if indicated. Monitor patients for any signs of hepatitis. *Rule out non-inflammatory causes of hepatotoxicity*: Infections, disease progression, or medications and monitor until resolution (e.g., at 3-day intervals). Provide appropriate symptomatic treatment and withhold or continue ipilimumab dosing as clinically appropriate	**AST or ALT >2.5x–5.0x ULN or total bilirubin >1.5x–3x ULN** (i) Withhold the dose of ipilimumab (ii) Check LFTs every day for 3 consecutive days; if LFT improvement to grade 1, resume routine monitoring of LFTs and continue ipilimumab (iii) If no improvement in the LFTs, administer corticosteroid treatment (e.g., prednisone 1-2 mg/kg/day) and continue to monitor closely (iv) Skip the next dose of ipilimumab until event resolves with no requirement for corticosteroids **AST or ALT >5x ULN or total bilirubin >3x ULN** (i) Permanently discontinue ipilimumab (ii) Treat with high-dose intravenous corticosteroid therapy (e.g., methylprednisolone 2 mg/kg qd or bid or equivalent) to control initial symptoms (iii) LFTs should be assessed daily (iv) If LFTs are stable or declining for 5 consecutive days, assess LFTs weekly until normalization; once symptoms have resolved and LFT elevations are normalized, initiate corticosteroid taper over ≥1 month; elevations in LFTs during taper may be managed with increase in corticosteroid dose or slower taper (v) If no response to corticosteroid therapy within 3–5 days, or for those with an LFT elevation during corticosteroid tapering that is not responsive to an increase in dose of corticosteroids, addition of immunosuppressive therapy with mycophenolate mofetil can be considered after a gastroenterology or hepatology consult (vi) Patients receiving immunosuppression for more than 4 weeks should be evaluated for prophylaxis of opportunistic infections per institutional guidelines

Ocular	Assess patients for uveitis, iritis or episcleritis	Administer corticosteroid drops with guidance from ophthalmology consultation; systemic corticosteroids should be considered in more severe cases

Neurologic	Encourage patient reporting of changes in muscle weakness or sensory alternations Patient may present with muscle weakness or sensory neuropathies lasting >5 days or motor neuropathies confirmed by physical examination *Rule out non-inflammatory causes*: Disease progression, infections (e.g., Lyme disease), metabolic syndromes, and medications (e.g., taxanes or platinum salts	**Grade 1**: **Asymptomatic, clinical observations only** (i) Treat symptoms per neurological consult recommendations (ii) Complete diagnostic testing: electromyogram and nerve conduction studies (iii) Continue ipilimumab (iv) Continue close monitoring **Grade 2**: **Moderate symptoms, limiting instrumental ADL** (i) Treat symptoms per neurological consult recommendations (ii) Complete diagnostic testing: electromyogram and nerve conduction studies (iii) Hold/delay ipilimumab until resolution to grade ≤1 **Grade 3 or 4**: **Severe symptoms, limiting self-case ADL, life-threatening consequences** (i) Treat symptoms per neurological consult recommendations (ii) Complete diagnostic testing: electromyogram and nerve conduction studies (iii) Consider intravenous corticosteroids (e.g., methylprednisolone 2 mg/kg qd or bid equivalent) (iv) Discontinue ipilimumab (v) If patient is not clinically stable or has atypical symptoms then hospitalize and administer IV corticosteroids and IV immunoglobulin or other immunosuppressive therapies as clinically appropriate

ALT: alanine aminotransferase; AST: aspartate aminotransferase; bid: twice daily; irAE: immune-related adverse event; IV: intravenous; LFTs: liver function tests; MRI: magnetic resonance imaging; PO: oral; qd: once daily; ULN: upper limit of normal.

## References

[1] van den Boorn JG, Konijnenberg D, Tjin EP (2010). Effective melanoma immunotherapy in mice by the skin-depigmenting agent monobenzone and the adjuvants imiquimod and CpG. *PloS ONE*.

[2] Sznol M (2009). Betting on immunotherapy for melanoma. *Current Oncology Reports*.

[3] Komenaka I, Hoerig H, Kaufman HL (2004). Immunotherapy for melanoma. *Clinics in Dermatology*.

[4] Gogas H, Ioannovich J, Dafni U (2006). Prognostic significance of autoimmunity during treatment of melanoma with interferon. *The New England Journal of Medicine*.

[5] Clemente CG, Mihm MC, Bufalino R, Zurrida S, Collini P, Cascinelli N (1996). Prognostic value of tumor infiltrating lymphocytes in the vertical growth phase of primary cutaneous melanoma. *Cancer*.

[6] Taylor RC, Patel A, Panageas KS, Busam KJ, Brady MS (2007). Tumor-infiltrating lymphocytes predict sentinel lymph node positivity in patients with cutaneous melanoma. *Journal of Clinical Oncology*.

[7] Håkansson A, Gustafsson B, Krysander L, Håkansson L (1996). Tumor-infiltrating lymphocytes in metastatic malignant melanoma and response to interferon alpha treatment. *The British Journal of Cancer*.

[8] Mihm MC, Clemente CG, Cascinelli N (1996). Tumor infiltrating lymphocytes in lymph node melanoma metastases: a histopathologic prognostic indicator and an expression of local immune response. *Laboratory Investigation*.

[9] Moschos SJ, Edington HD, Land SR (2006). Neoadjuvant treatment of regional stage IIIB melanoma with high-dose interferon alfa-2b induces objective tumor regression in association with modulation of tumor infiltrating host cellular immune responses. *Journal of Clinical Oncology*.

[10] Mellman I, Coukos G, Dranoff G (2011). Cancer immunotherapy comes of age. *Nature*.

[11] Weber JS, Kähler KC, Hauschild A (2012). Management of immune-related adverse events and kinetics of response with ipilimumab. *Journal of Clinical Oncology*.

[12] Phan GQ, Yang JC, Sherry RM (2003). Cancer regression and autoimmunity induced by cytotoxic T lymphocyte-associated antigen 4 blockade in patients with metastatic melanoma. *Proceedings of the National Academy of Sciences of the United States of America*.

[13] Maker AV, Phan GQ, Attia P (2005). Tumor regression and autoimmunity in patients treated with cytotoxic T lymphocyte-associated antigen 4 blockade and interleukin 2: a phase I/II study. *Annals of Surgical Oncology*.

[14] Carpenter KJ, Murtagh RD, Lilienfeld H, Weber J, Murtagh FR (2009). Ipilimumab-induced hypophysitis: MR imaging findings. *The American Journal of Neuroradiology*.

[15] Attia P, Phan GQ, Maker AV (2005). Autoimmunity correlates with tumor regression in patients with metastatic melanoma treated with anti-cytotoxic T-lymphocyte antigen-4. *Journal of Clinical Oncology*.

[16] Hodi FS, O’Day SJ, McDermott DF (2010). Improved survival with ipilimumab in patients with metastatic melanoma. *The New England Journal of Medicine*.

[17] Robert C, Thomas L, Bondarenko I (2011). Ipilimumab plus dacarbazine for previously untreated metastatic melanoma. *The New England Journal of Medicine*.

[18] Wolchok J, Neyns B, Linette G (2010). Ipilimumab monotherapy in patients with previously treated, advanced melanoma: a randomized, double-blind, multicenter, phase 2, dose-ranging study. *The Lancet Oncology*.

[19] Weber J, Thompson JA, Hamid O (2009). A randomized, double-blind, placebo-controlled, phase II study comparing the tolerability and efficacy of ipilimumab administered with or without prophylactic budesonide in patients with unresectable stage III or IV melanoma. *Clinical Cancer Research*.

[20] O’Day SJ, Maio M, Chiarion-Sileni V (2010). Efficacy and safety of ipilimumab monotherapy in patients with pretreated, advanced melanoma: a multicenter single-arm phase II study. *Annals of Oncology*.

[21] Hersh EM, O’Day SJ, Powderly J (2011). A phase II multicenter study of ipilimumab with or without dacarbazine in chemotherapy-naïve patients with advanced melanoma. *Investigational New Drugs*.

[22] Pardoll DM (2012). The blockade of immune checkpoints in cancer immunotherapy. *Nature Reviews Cancer*.

[23] Peggs KS, Quezada SA (2010). Ipilimumab: attenuation of an inhibitory immune checkpoint improves survival in metastatic melanoma. *Expert Review of Anticancer Therapy*.

[24] Ibrahim R, Berman D, de Pril V V (2011). Ipilimumab safety profile: summary of findings from completed trials in advanced melanoma. *Journal of Clinical Oncology*.

[25] Dummer R, Maio M, Hamid O Time to onset and resolution of immune-related adverse events associated with ipilimumab therapy in patients with advanced melanoma.

[26] YERVOY (ipilimumab): serious and fatal immune-mediated adverse reactions. http://www.yervoy.com/hcp/rems.aspx.

[27] Tarhini AA, Kirkwood JM (2010). CTLA-4-blocking immunotherapy with ipilimumab for advanced melanoma. *Oncology*.

[28] Korn EL, Liu PY, Lee SJ (2008). Meta-analysis of phase II cooperative group trials in metastatic stage IV melanoma to determine progression-free and overall survival benchmarks for future phase II trials. *Journal of Clinical Oncology*.

[29] Balch CM, Gershenwald JE, Soong SJ (2009). Final version of 2009 AJCC melanoma staging and classification. *Journal of Clinical Oncology*.

[30] Kirkwood JM, Tarhini AA, Panelli MC (2008). Next generation of immunotherapy for melanoma. *Journal of Clinical Oncology*.

[31] Agarwala SS (2009). Current systemic therapy for metastatic melanoma. *Expert Review of Anticancer Therapy*.

[32] Antony GK, Dudek AZ (2010). Interleukin 2 in cancer therapy. *Current Medicinal Chemistry*.

[33] Kirkwood JM, Butterfield LH, Tarhini AA, Zarour H, Kalinski P, Ferrone S (2012). Immunotherapy of cancer in 2012. *CA: A Cancer Journal for Clinicians*.

[34] Thumar JR, Kluger HM (2010). Ipilimumab: a promising immunotherapy for melanoma. *Oncology*.

[35] Maker AV, Yang JC, Sherry RM (2006). Intrapatient dose escalation of anti-CTLA-4 antibody in patients with metastatic melanoma. *Journal of Immunotherapy*.

[36] Weber JS, O’Day S, Urba W (2008). Phase I/II study of ipilimumab for patients with metastatic melanoma. *Journal of Clinical Oncology*.

[37] Weber J (2009). Ipilimumab: controversies in its development, utility, and autoimmune adverse events. *Cancer Immunology, Immunotherapy*.

[38] Rivoltini L, Carrabba M, Huber V (2002). Immunity to cancer: attack and escape in T lymphocyte-tumor cell interaction. *Immunological Reviews*.

[39] Melero I, Hervas-Stubbs S, Glennie M, Pardoll DM, Chen L (2007). Immunostimulatory monoclonal antibodies for cancer therapy. *Nature Reviews Cancer*.

[40] Hoos A, Ibrahim R, Korman A (2010). Development of ipilimumab: contribution to a new paradigm for cancer immunotherapy. *Seminars in Oncology*.

[41] Weber J (2010). Immune checkpoint proteins: a new therapeutic paradigm for cancer—preclinical background: CTLA-4 and PD-1 blockade. *Seminars in Oncology*.

[42] Boasberg P, Hamid O, O’Day S (2010). Ipilimumab: unleashing the power of the immune system through CTLA-4 blockade. *Seminars in Oncology*.

[43] Nagorsen D, Scheibenbogen C, Marincola FM, Letsch A, Keilholz U (2003). Natural T cell immunity against cancer. *Clinical Cancer Research*.

[44] Dunn GP, Bruce AT, Ikeda H, Old LJ, Schreiber RD (2002). Cancer immunoediting: from immunosurveillance to tumor escape. *Nature Immunology*.

[45] Shankaran V, Ikeda H, Bruce AT (2001). IFN*γ*, and lymphocytes prevent primary tumour development and shape tumour immunogenicity. *Nature*.

[46] DuPage M, Mazumdar C, Schmidt LM, Cheung AF, Jacks T (2012). Expression of tumour-specific antigens underlies cancer immunoediting. *Nature*.

[47] Pandolfi F, Cianci R, Lolli S (2008). Strategies to overcome obstacles to successful immunotherapy of melanoma. *International Journal of Immunopathology and Pharmacology*.

[48] Kurnick JT, Ramirez-Montagut T, Boyle LA (2001). A novel autocrine pathway of tumor escape from immune recognition: melanoma cell lines produce a soluble protein that diminishes expression of the gene encoding the melanocyte lineage Melan-A/MART-1 antigen through down-modulation of its promoter. *Journal of Immunology*.

[49] Gabrilovich DI, Nagaraj S (2009). Myeloid-derived suppressor cells as regulators of the immune system. *Nature Reviews Cancer*.

[50] Pandolfi F, Cianci R, Pagliari D (2011). The immune response to tumors as a tool toward immunotherapy. *Clinical and Developmental Immunology*.

[51] Alexandrescu DT, Ichim TE, Riordan NH (2010). Immunotherapy for melanoma: current status and perspectives. *Journal of Immunotherapy*.

[52] Wang W, Lau R, Yu D, Zhu W, Korman A, Weber J (2009). PD1 blockade reverses the suppression of melanoma antigen-specific CTL by CD4^+^CD25Hi regulatory T cells. *International Immunology*.

[53] Engelhardt JJ, Sullivan TJ, Allison JP (2006). CTLA-4 overexpression inhibits T cell responses through a CD28-B7-dependent mechanism. *Journal of Immunology*.

[54] Keler T, Halk E, Vitale L (2003). Activity and safety of CTLA-4 blockade combined with vaccines in cynomolgus macaques. *Journal of Immunology*.

[55] Robert C, Ghiringhelli F (2009). What is the role of cytotoxic T lymphocyte-associated antigen 4 blockade in patients with metastatic melanoma?. *Oncologist*.

[56] Weber JS, Hamid O, Chasalow SD (2012). Ipilimumab increases activated T cells and enhances humoral immunity in patients with advanced melanoma. *Journal of Immunotherapy*.

[57] Brahmer JR, Tykodi SS, Chow LQ (2012). Safety and activity of anti-PD-L1 antibody in patients with advanced cancer. *The New England Journal of Medicine*.

[58] Schadendorf D, Algarra SM, Bastholt L (2009). Immunotherapy of distant metastatic disease. *Annals of Oncology*.

[59] Li B, Lin J, VanRoey M, Jure-Kunkel M, Jooss K (2007). Established B16 tumors are rejected following treatment with GM-CSF-secreting tumor cell immunotherapy in combination with anti-4-1BB mAb. *Clinical Immunology*.

[60] Choi BK, Kim YH, Kang WJ (2007). Mechanisms involved in synergistic anticancer immunity of anti-4-1BB and anti-CD4 therapy. *Cancer Research*.

[61] Sznol M, Hodi FS, Margolin K (2008). Phase I study of BMS-663513, a fully human anti-CD137 agonist monoclonal antibody, in patients (pts) with advanced cancer (CA). 2008 ASCO annual proceedings part I. *Journal of Clinical Oncology*.

[62] Wolchok JD, Weber JS, Hamid O (2010). Ipilimumab efficacy and safety in patients with advanced melanoma: a retrospective analysis of HLA subtype from four trials. *Cancer Immunity*.

[63] Farolfi A, Ridolfi L, Guidoboni M (2012). Ipilimumab in advanced melanoma: reports of long-lasting responses. *Melanoma Research*.

[64] Prieto PA, Yang JC, Sherry RM (2012). CTLA-4 blockade with ipilimumab: long-term follow-up of 177 patients with metastatic melanoma. *Clinical Cancer Research*.

[65] Heller K, Pavlick AC, Hodi FS (2011). Safety and survival analysis of ipilimumab therapy in patients with stable asymptomatic brain metastases. *Journal of Clinical Oncology*.

[66] Margolin K, Ernstoff MS, Hamid O (2012). Ipilimumab in patients with melanoma and brain metastases: an open-label, phase 2 trial. *The Lancet Oncology*.

[67] Wolchok JD, Hoos A, O’Day S (2009). Guidelines for the evaluation of immune therapy activity in solid tumors: immune-related response criteria. *Clinical Cancer Research*.

[68] Hoos A, Eggermont AM, Janetzki S (2010). Improved endpoints for cancer immunotherapy trials. *Journal of the National Cancer Institute*.

[69] Di Giacomo AM, Biagioli M, Maio M (2010). The emerging toxicity profiles of antiCTLA-4 antibodies across clinical indications. *Seminars in Oncology*.

[70] Kaehler KC, Piel S, Livingstone E, Schilling B, Hauschild A, Schadendorf D (2010). Update on immunologic therapy with antiCTLA-4 antibodies in melanoma: identification of clinical and biological response patterns, immune-related adverse events, and their management. *Seminars in Oncology*.

[71] Read S, Greenwald R, Izcue A (2006). Blockade of CTLA-4 on CD4^+^CD25^+^ regulatory T cells abrogates their function in vivo. *Journal of Immunology*.

[72] Korman AJ, Peggs KS, Allison JP (2006). Checkpoint blockade in cancer immunotherapy. *Advances in Immunology*.

[73] Féaux de Lacroix W, Runne U, Hauk H, Doepfmer K, Groth W, Wacker D (1983). Acute liver dystrophy with thrombosis of hepatic veins: a fatal complication of dacarbazine treatment. *Cancer Treatment Reports*.

[74] Horiguchi M, Kim J, Matsunaga N (2010). Glucocorticoid-dependent expression of O6-methylguanine-DNA methyltransferase gene modulates dacarbazine-induced hepatotoxicity in mice. *Journal of Pharmacology and Experimental Therapeutics*.

[75] YERVOY (ipilimumab) Injection for intravenous infusion. http://packageinserts.bms.com/pi/pi_yervoy.pdf.

[76] Patel SP, Woodman SE (2011). Profile of ipilimumab and its role in the treatment of metastatic melanoma. *Drug Design, Development and Therapy*.

[77] Lebbé C, O’Day SJ, Sileni VC Analysis of the onset and resolution of immune-related adverse events during treatment with ipilimumab in patients with metastatic melanoma.

[78] Lacouture ME, Melosky BL (2007). Cutaneous reactions to anticancer agents targeting the epidermal growth factor receptor: a dermatology-oncology perspective. *Skin Therapy Letter*.

[79] Luu M, Boone SL, Patel J (2011). Higher severity grade of erlotinib-induced rash is associated with lower skin phototype. *Clinical and Experimental Dermatology*.

[80] Jaber SH, Cowen EW, Haworth LR (2006). Skin reactions in a subset of patients with stage IV melanoma treated with anti-cytotoxic T-lymphocyte antigen 4 monoclonal antibody as a single agent. *Archives of Dermatology*.

[81] Pavlick AC, Ott PA, Kannan K (2010). Hair depigmentation as an indicator of a durable response to CTLA-4 therapy. *Journal of Clinical Oncology*.

[82] Oble DA, Mino-Kenudson M, Goldsmith J (2008). Alpha-CTLA-4 mAb-associated panenteritis: a histologic and immunohistochemical analysis. *The American Journal of Surgical Pathology*.

[83] Berman D, Parker SM, Siegel J (2010). Blockade of cytotoxic T-lymphocyte antigen-4 by ipilimumab results in dysregulation of gastrointestinal immunity in patients with advanced melanoma. *Cancer Immunity*.

[84] Beck KE, Blansfield JA, Tran KQ (2006). Enterocolitis in patients with cancer after antibody blockade of cytotoxic T-lymphocyte-associated antigen 4. *Journal of Clinical Oncology*.

[85] Minor DR, Chin K, Kashani-Sabet M (2009). Infliximab in the treatment of anti-CTLA4 antibody (ipilimumab) induced immune-related colitis. *Cancer Biotherapy and Radiopharmaceuticals*.

[86] Lord JD, Hackman RC, Moklebust A (2010). Refractory colitis following anti-CTLA4 antibody therapy: analysis of mucosal FOXP3+ T cells. *Digestive Diseases and Sciences*.

[87] Peggs KS, Quezada SA, Korman AJ, Allison JP (2006). Principles and use of anti-CTLA4 antibody in human cancer immunotherapy. *Current Opinion in Immunology*.

[88] Phan GQ, Weber JS, Sondak VK (2008). CTLA-4 blockade with monoclonal antibodies in patients with metastatic cancer: surgical issues. *Annals of Surgical Oncology*.

[89] Freeman HJ (2012). Colitis associated with biological agents. *World Journal of Gastroenterology*.

[90] Smith FO, Goff SL, Klapper JA (2007). Risk of bowel perforation in patients receiving interleukin-2 after therapy with anti-CTLA 4 monoclonal antibody. *Journal of Immunotherapy*.

[91] Torino F, Barnabei A, de Vecchis L, Salvatori R, Corsello SM (2012). Hypophysitis induced by monoclonal antibodies to cytotoxic T lymphocyte antigen 4: challenges from a new cause of a rare disease. *The Oncologist*.

[92] Juszczak A, Gupta A, Karavitaki N, Middleton MR, Grossman AB (2012). Ipilimumab: a novel immunomodulating therapy causing autoimmune hypophysitis: a case report and review. *European Journal of Endocrinology*.

[93] Thurmar JR, Kluger HM (2010). Ipilimumab: a promising immunotherapy for melanoma. *Oncology*.

[94] Min L, Vaidya A, Becker C (2011). Thyroid autoimmunity and ophthalmopathy related to melanoma biological therapy. *European Journal of Endocrinology*.

[95] Borodic G, Hinkle DM, Cia Y (2011). Drug-induced graves disease from CTLA-4 receptor suppression. *Ophthalmic Plastic and Reconstructive Surgery*.

[96] Hunter G, Voll C, Robinson CA (2009). Autoimmune inflammatory myopathy after treatment with ipilimumab. *Canadian Journal of Neurological Sciences*.

[97] Yang JC, Hughes M, Kammula U (2007). Ipilimumab (anti-CTLA4 antibody) causes regression of metastatic renal cell cancer associated with enteritis and hypophysitis. *Journal of Immunotherapy*.

[98] Bompaire F, Mateus C, Taillia H (2012). Severe meningo-radiculo-nevritis associated with ipilimumab. *Investigational New Drugs*.

[99] Wilgenhof S, Neyns B (2011). Anti-CTLA-4 antibody-induced Guillain-Barré syndrome in a melanoma patient. *Annals of Oncology*.

[100] Bhatia S, Huber BR, Upton MP, Thompson JA (2009). Inflammatory enteric neuropathy with severe constipation after ipilimumab treatment for melanoma: a case report. *Journal of Immunotherapy*.

[101] Maur M, Tomasello C, Frassoldati A, Dieci MV, Barbieri E, Conte P (2012). Posterior reversible encephalopathy syndrome during ipilimumab therapy for malignant melanoma. *Journal of Clinical Oncology*.

[102] Di Giacomo AM, Danielli R, Guidoboni M (2009). Therapeutic efficacy of ipilimumab, an anti-CTLA-4 monoclonal antibody, in patients with metastatic melanoma unresponsive to prior systemic treatments: clinical and immunological evidence from three patient cases. *Cancer Immunology, Immunotherapy*.

[103] Gordon IO, Wade T, Chin K, Dickstein J, Gajewski TF (2009). Immune-mediated red cell aplasia after anti-CTLA-4 immunotherapy for metastatic melanoma. *Cancer Immunology, Immunotherapy*.

[104] Di Giacomo AM, Danielli R, Calabrò L (2011). Ipilimumab experience in heavily pretreated patients with melanoma in an expanded access program at the University Hospital of Siena (Italy). *Cancer Immunology, Immunotherapy*.

[105] Akhtari M, Waller EK, Jaye DL (2009). Neutropenia in a patient treated with ipilimumab (anti-CTLA-4 Antibody). *Journal of Immunotherapy*.

[106] Wilgenhof S, Morlion V, Seghers AC Sarcoidosis in a patient with metastatic melanoma sequentially treated with anti-CTLA-4 monoclonal antibody and selective BRAF inhibitor. *Anticancer Research*.

[107] Vogel WV, Guislain A, Kvistborg P, Schumacher TN, Haanen JB, Blank CU (2012). Ipilimumab-induced sarcoidosis in a patient with metastatic melanoma undergoing complete remission. *Journal of Clinical Oncology*.

[108] Eckert A, Schoeffler A, Dalle S, Phan A, Kiakouama L, Thomas L (2009). Anti-CTLA4 monoclonal antibody induced sarcoidosis in a metastatic melanoma patient. *Dermatology*.

[109] Fadel F, El Karoui K, Knebelmann B (2009). Anti-CTLA4 antibody-induced lupus nephritis. *The New England Journal of Medicine*.

[110] Delyon J, Mateus C, Lambert T (2011). Hemophilia A induced by ipilimumab. *The New England Journal of Medicine*.

[111] Lozier J (2012). More on hemophilia A induced by ipilimumab. *The New England Journal of Medicine*.

[112] Lutzky J, Wolchok J, Hamid O (2009). Association between immune-related adverse events (irAEs) and disease control or overall survival in patients (pts) with advanced melanoma treated with 10 mg/kg ipilimumab in three phase II clinical trials. *Journal of Clinical Oncology*.

[113] Tarhini AA, Cherian J, Moschos SJ (2012). Safety and efficacy of combination immunotherapy with interferon alfa-2b and tremelimumab in patients with stage IV melanoma. *Journal of Clinical Oncology*.

[114] Bronstein Y, Ng CS, Hwu P, Hwu WJ (2011). Radiologic manifestations of immune-related adverse events in patients with metastatic melanoma undergoing anti-CTLA-4 antibody therapy. *The American Journal of Roentgenology*.

[115] Ji RR, Chasalow SD, Wang L (2012). An immune-active tumor microenvironment favors clinical response to ipilimumab. *Cancer Immunology, Immunotherapy*.

[116] O’Day SJ, Hamid O, Urba WJ (2007). Targeting cytotoxic T-lymphocyte antigen-4 (CTLA-4): a novel strategy for the treatment of melanoma and other malignancies. *Cancer*.

[117] Amin A, DePril V, Hamid O (2009). Evaluation of the effect of systemic corticosteroids for the treatment of immune-related adverse events (irAEs) on the development or maintenance of ipilimumab clinical activity. *Journal of Clinical Oncology*.

[118] Harmankaya K, Erasim C, Koelblinger C (2011). Continuous systemic corticosteroids do not affect the ongoing regression of metastatic melanoma for more than two years following ipilimumab therapy. *Medical Oncology*.

[119] Grob JJ, Hamid O, Wolchok J Antitumor responses to ipilimumab in advanced melanoma are not affected by systemic corticosteroids used to manage immune-related adverse events (irAEs).

[120] Downey SG, Klapper JA, Smith FO (2007). Prognostic factors related to clinical response in patients with metastatic melanoma treated by CTL-associated antigen-4 blockade. *Clinical Cancer Research*.

[121] Lemech C, Arkenau HT (2012). Novel treatments for metastatic cutaneous melanoma and the management of emergent toxicities. *Clinical Medicine Insights, Oncology*.

[122] Tarhini A, Lo E, Minor DR (2010). Releasing the brake on the immune system: ipilimumab in melanoma and other tumors. *Cancer Biotherapy and Radiopharmaceuticals*.

[123] Verschraegen C (2012). . The monoclonal antibody to cytotoxic T lymphocyte antigen 4, ipilimumab, in the treatment of melanoma. *Cancer Management and Research*.

[124] Rubin KM (2012). Managing immune-related adverse events to ipilimumab: a nurse's guide. *Clinical Journal of Oncology Nursing*.

[125] O’Day S, Weber JS, Wolchok JD (2011). Effectiveness of treatment guidance on diarrhea and colitis across ipilimumab studies. *Journal of Clinical Oncology*.

[126] Kaehler KC, Egberts F, Lorigan P, Hauschilda A (2009). Anti-CTLA-4 therapy-related autoimmune hypophysitis in a melanoma patient. *Melanoma Research*.

[127] Chmiel KD, Suan D, Liddle C (2011). Resolution of severe ipilimumab-induced hepatitis after antithymocyte globulin therapy. *Journal of Clinical Oncology*.

[128] REMICADE (infliximab) Lyophilized concentrate for injection, for intravenous use. http://www.remicade.com/remicade/assets/hcp_ppi.pdf.

[129] Tarhini AA, Iqbal F (2010). CTLA-4 blockade: therapeutic potential in cancer treatments. *OncoTargets and Therapy*.

